# Real-world use of emapalumab in hemophagocytic lymphohistiocytosis: a scoping review of published evidence

**DOI:** 10.3389/fphar.2026.1749084

**Published:** 2026-04-10

**Authors:** Abdulrahman F. Al-Mashdali, Fatihelmgib Mohamed, Marwa Osman, Mujahid O. Abdelraof, Mona Al Rasheed, Hind Salama, Shehab F. Mohamed, Mohamed A. Yassin

**Affiliations:** 1 Department of Hematology, National Center for Cancer Care and Research, Hamad Medical Corporation, Doha, Qatar; 2 Department of Public and Environmental Health, Faculty of Medicine, Gezira University, Wad Madani, Sudan; 3 Faculty of Medicine, Shendi University, Shendi, Sudan; 4 Clinical Hematology Department, Adan Hospital, Al Ahmadi, Kuwait; 5 Adult Hematology Department, King Abdulaziz Medical City, Riyadh, Saudi Arabia

**Keywords:** cytokine release syndrome, emapalumab, hemophagocytic lymphohistiocytosis, interferon-γ blockade, macrophageactivation syndrome, real-world evidence

## Abstract

**Background:**

Emapalumab, an interferon-γ (interferon-gamma)–blocking monoclonal antibody, has emerged as a targeted therapy for refractory hemophagocytic lymphohistiocytosis (HLH). This scoping review summarizes real-world evidence of its clinical use across HLH subtypes.

**Methods:**

A comprehensive search of PubMed, Scopus, and Web of Science through September 2025 identified studies reporting emapalumab use outside clinical trials. Case reports, series, and observational studies describing clinical outcomes were included.

**Results:**

Thirty-one publications comprising 86 patients were analyzed. Disease contexts included familial/genetic HLH (n = 11), rheumatology-associated macrophage activation syndrome (MAS; n = 12), malignancy-associated HLH (n = 25), infection-associated HLH (n = 22), CAR-T/IEC-HS or cytokine-release-syndrome–related HLH (chimeric antigen receptor T-cell/immune effector cell–associated hemophagocytic syndrome; n = 11), and other secondary HLH (n = 5). Across all categories, emapalumab achieved rapid suppression of hyperinflammation, typically within 1–2 weeks. Clinical response rates were 100% in familial HLH, 91.7% in rheumatology-associated MAS, 72.7% in infection-associated HLH, 90.9% in CAR-T/IEC-HS–related HLH, and 80% in other secondary HLH. In contrast, only 6 of 25 patients with malignancy-associated HLH (24%) showed partial or complete responses, reflecting inferior outcomes due to underlying disease progression. Overall survival was highest in familial and infection-associated subgroups. The reported adverse events were generally infrequent and mild in the published cases, but causality cannot be reliably established given the complexity of the underlying disease and concurrent therapies.

**Conclusion:**

Real-world evidence demonstrates that emapalumab induces rapid and durable disease control across diverse HLH subtypes, with a favorable tolerability profile. However, outcomes remain markedly inferior in malignancy-associated HLH, where response rates are limited to approximately 24%, primarily due to the impact of underlying malignancy. Its role as rescue or bridging therapy to hematopoietic stem-cell transplantation (HSCT) is increasingly supported in both pediatric and adult populations.

## Introduction

Hemophagocytic lymphohistiocytosis (HLH) is a life-threatening hyperinflammatory syndrome marked by uncontrolled immune activation, a cytokine surge, and progressive multi-organ dysfunction. It includes familial or genetic HLH, driven by defects in cytotoxic lymphocyte function, and secondary HLH that complicates rheumatologic disease, malignancy, infection, or modern cellular immunotherapy (La Rosée et al., 2019). Within the latter, immune effector cell-associated HLH-like syndrome (IEC-HS) and severe cytokine release syndrome (CRS) after chimeric antigen receptor T-cell therapy have emerged as highly morbid states. Recent consensus criteria from the American Society for Transplantation and Cellular Therapy (ASTCT) help distinguish IEC-HS from overlapping toxicities and guide initial management (Hines et al., 2023). Typical features of HLH include persistent fever, hepatosplenomegaly, lymphadenopathy, and cytopenias, accompanied by laboratory evidence of hyperinflammation such as marked hyperferritinemia, hypertriglyceridemia, elevated IL-2 levels, and hypofibrinogenemia. These manifestations often progress to multiorgan dysfunction and may include neurologic involvement. Diagnosis usually relies on the HLH-2004 criteria and the HScore. HLH is classified as primary when a pathogenic genetic defect is present and as secondary when no causative genetic variant is identified (Ponnatt et al., 2022). Management of HLH centers on rapid recognition, treatment of the trigger, and swift control of hyperinflammation. The standard backbone remains high-dose dexamethasone with etoposide, with cyclosporine and intrathecal therapy for CNS involvement, and hematopoietic stem-cell transplantation for primary or relapsing disease. In adults and secondary HLH, care is individualized: early antimicrobials, targeted therapy for EBV when appropriate, and definitive treatment of malignancy run in parallel with immunosuppression. Targeted agents are used when standard therapy is refractory or intolerable, including anakinra and ruxolitinib (Henter, 2025).

Interferon gamma is a central driver of HLH biology. It amplifies macrophage activation, antigen presentation, and chemokine networks that sustain hemophagocytosis and tissue injury (Brisse et al., 2015). Emapalumab, a fully human monoclonal antibody that specifically binds to and neutralizes interferon gamma (IFN-γ), was approved by the U.S. Food and Drug Administration (FDA) in 2018 for the treatment of primary hemophagocytic lymphohistiocytosis (HLH) that is refractory, recurrent, progressive, or intolerant to conventional therapy. This approval marked the first targeted immunotherapy directed at a key cytokine implicated in HLH pathogenesis, offering a mechanistically precise alternative to traditional cytotoxic and broadly immunosuppressive regimens (Vallurupalli and Berliner, 2019). Of note, a potential advantage of IFN-γ blockade therapy for primary HLH may be its ability to improve engraftment in allogeneic HSCT, preventing and treating graft failure (Wu et al., 2024). On 27 June 2025, the U.S. Food and Drug Administration (FDA) approved emapalumab for the treatment of macrophage activation syndrome (MAS) in patients with known or suspected Still’s disease, including both adult-onset Still’s disease (AOSD) and systemic juvenile idiopathic arthritis (sJIA), who have shown an inadequate response or intolerance to glucocorticoids (Benedetti et al., 2023). Real-world use of emapalumab has expanded to encompass both primary and secondary disease contexts, including rheumatologic flares, infection-triggered HLH such as Epstein–Barr virus–associated cases, malignancy-associated HLH, and IEC-HS or refractory CRS following cellular therapy. In addition, emapalumab has recently been employed for the treatment of immune-mediated graft failure after hematopoietic stem-cell transplantation (HSCT) (Johnson et al., 2024; Wang et al., 2024; Schuelke et al., 2023; Merli et al., 2025).

Despite increasing bedside experience, evidence outside clinical trials remains fragmented across case reports, small series, and retrospective cohorts, with notable heterogeneity in patient selection, dosing regimens, concomitant immunomodulation, and outcome definitions. Accordingly, we conducted a scoping review focused on peer-reviewed, real-world evidence regarding the use of emapalumab outside interventional protocols across major HLH contexts, including IEC-HS and CRS following cellular therapy. This review systematically aggregated data on study design, patient characteristics, dosing strategies, concomitant therapies, response kinetics, bridging to hematopoietic stem-cell transplantation, relapse, survival, and safety. Harmonized evidence tables and a narrative synthesis were developed to integrate diverse clinical experiences into a unified framework aimed at supporting urgent, high-stakes therapeutic decisions at the bedside.

## Methods

### Search strategy

A comprehensive scoping review was conducted to evaluate the real-world use of *emapalumab* outside interventional clinical trial settings. Searches were performed across PubMed/MEDLINE, Scopus, and Web of Science from database inception to 30 September 2025, using combinations of keywords and MeSH terms, including *“emapalumab”*, *“interferon gamma blockade”*, *“HLH”*, *“hemophagocytic lymphohistiocytosis”*, *“MAS”*, *“macrophage activation syndrome”*, *“CAR-T”*, *“IEC-HS”*, *“malignancy-associated HLH”*, and *“infection-associated HLH.”* Reference lists of all eligible studies were hand-searched to identify additional relevant publications.

### Eligibility criteria

Studies were eligible if they met the following inclusion criteria:

1. Population: Patients of any age with HLH or HLH-like hyperinflammatory syndromes, including familial/genetic, secondary, rheumatologic, infection-associated, malignancy-associated, CAR-T/CRS-related, or idiopathic forms.
2. Intervention: Administration of emapalumab in any dosing regimen or duration.
3. Setting: Real-world clinical contexts, including case reports, case series, and observational studies.
4. Outcomes: Any clinical, laboratory, or safety outcomes related to emapalumab use.

Exclusion criteria clinical trials, preclinical or animal studies, conference abstracts lacking sufficient detail, review articles without original patient data, duplicate or overlapping publications, and non-English language studies.

### Study selection

**FIGURE 1:**
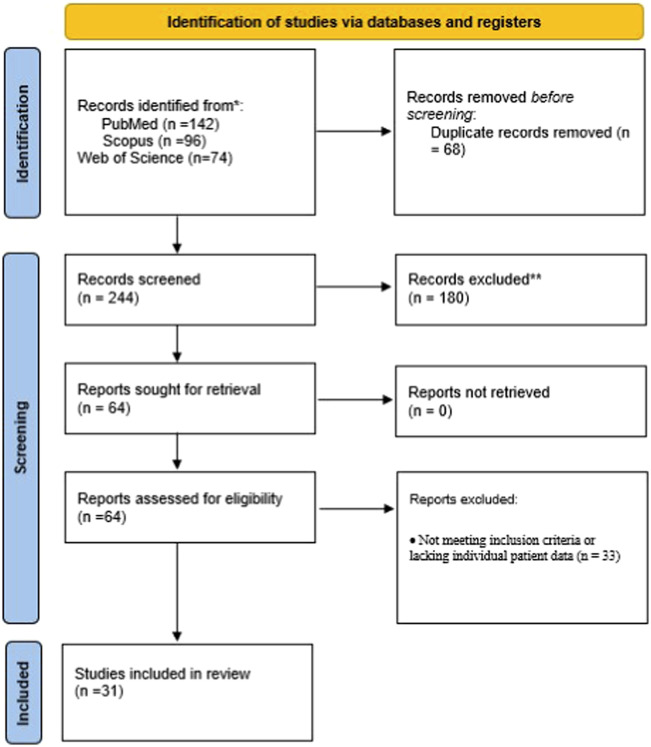
The PRISMA-ScR flow diagram detailing the study selection process.

Two independent reviewers screened titles and abstracts for relevance. Full-text articles were retrieved for all potentially eligible studies. Discrepancies were resolved by discussion or adjudication by a third reviewer. The selection process followed the PRISMA-ScR (Preferred Reporting Items for Systematic Reviews and Meta-Analyses Extension for Scoping Reviews) guidelines and is summarized in Figure 1.

### Data extraction

Data were independently extracted using a standardized form capturing study characteristics (author, year, country, design), patient demographics, HLH etiology, diagnostic criteria, emapalumab dosing and duration, concomitant therapies, clinical outcomes (response, remission, HSCT), follow-up duration, and adverse events. Where available, survival and relapse data were also collected.

### Data synthesis

Findings were synthesized qualitatively and grouped according to the underlying HLH etiology: familial/genetic, infection-associated, malignancy-associated, rheumatologic/MAS, CAR-T/CRS-associated, and other secondary causes. Descriptive statistics (counts, ranges, medians) were reported where appropriate. Due to heterogeneity in study design, population, and outcome definitions, quantitative meta-analysis was not performed.

## Results

### Search results

A total of 312 records were identified through database searches across PubMed, Scopus, and Web of Science. After removal of duplicates, 244 records were screened by title and abstract. Of these, 64 full-text articles were reviewed for eligibility, and 31 studies met the inclusion criteria. In total, 86 unique patients treated with *emapalumab* for hemophagocytic lymphohistiocytosis (HLH) or related hyperinflammatory syndromes were identified. The PRISMA-ScR flow diagram summarizing the study selection process is presented in Figure 1.

**FIGURE 2:**
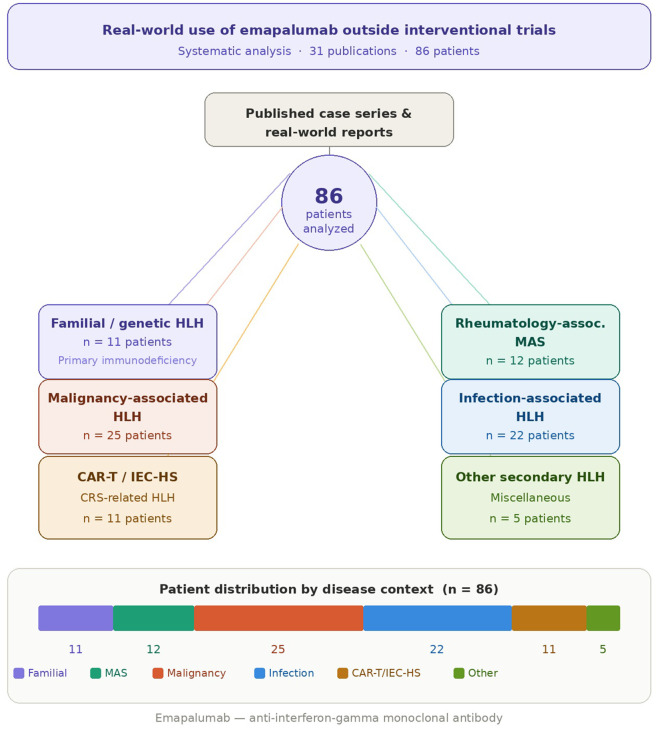
Data represents the clinical use of emapalumab compiled from published real-world reports outside interventional trial settings.

Figure 2 illustrates the distribution of patients by HLH etiology, highlighting that all cases represent the real-world use of *emapalumab* compiled from published clinical reports outside interventional trial settings.

Of note, except for hereditary/familial HLH, all reported uses of emapalumab in this review were off-label, including use in MAS prior to regulatory approval.

### Breakdown by disease context

- Familial/Genetically Confirmed HLH (fHLH): Eleven patients across five publications (Merli et al., 2019; Lam et al., 2019; Alahmari and Khogeer, 2023; Schuelke et al., 2023; Hahn et al., 2024).
- Rheumatology-Associated Macrophage Activation Syndrome (MAS): Twelve patients from eight reports (Gabr et al., 2020; Guild et al., 2022; Schuelke et al., 2023; Song et al., 2024; Slaney et al., 2024; Xu et al., 2025; Faggioli et al., 2025; Macaraeg and Schulert, 2023).
- Malignancy-Associated HLH (M-HLH): Twenty-five patients across six publications (Johnson et al., 2024; Song et al., 2024; Krishnareddigari et al., 2025; Maher et al., 2025; Schuelke et al., 2023; Goldstein et al., 2023).
- Infection-Associated HLH: Twenty-two patients from eleven reports (Dupuy et al., 2020; Jia et al., 2024; Lounder et al., 2019; McKeone et al., 2022; Schuelke et al., 2023; Song et al., 2024; Squire et al., 2020; Triebwasser et al., 2021; Tucci et al., 2021; Wang et al., 2024; Zhu et al., 2025).
- CAR-T/IEC-HS or Cytokine Release Syndrome (CRS)-Related HLH: Eleven patients across six studies (McNerney et al., 2022; Rainone et al., 2023; Schuelke et al., 2023; Scala et al., 2024; Ruemmele et al., 2025; Manghisi et al., 2025).
- Other Secondary HLH Five patients across four reports (Guild et al., 2022; Schuelke et al., 2023; Song et al., 2024; Wu et al., 2025).

### Emapalumab in familial HLH

Across five published reports including eleven children with familial or genetically confirmed HLH, emapalumab was primarily used in patients with refractory or relapsing disease (Schuelke et al., 2023; Alahmari and Khogeer, 2023; Hahn et al., 2024; Merli et al., 2019; Lam et al., 2019) (Table 1). Most were infants and young children under 2 years of age, though cases ranged from the neonatal period to early childhood. Genetic testing identified pathogenic variants in UNC13D, STXBP2, and PRF1, including both homozygous and compound heterozygous mutations. One patient had Chédiak–Higashi syndrome due to a LYST mutation, and another had NOCARH syndrome linked to a CDC42 abnormality. All patients met diagnostic criteria for HLH or had a clinical presentation consistent with fHLH. The HLH-2004 criteria were most frequently applied, while some genetically confirmed cases were classified as HLH without additional scoring.

**TABLE 1:** Study characteristics, treatment details, and clinical outcomes of patients with familial or genetic hemophagocytic lymphohistiocytosis (HLH) treated with emapalumab.

No.	Author/Year	Country	Study design	Age	Sex	HLH etiology	Genetic mutation	Prior treatment before emapalumab	Indication for starting	Emapalumab dose	Duration	Total doses
1	Merli et al. (2019)	Italy	Prospective study	11 months	M	Familial HLH	UNC13D	HSCT, steroids	Graft failure in HLH	6 mg/kg	24 days	8
2	Merli et al. (2019)	Italy	Prospective study	12 months	F	Familial HLH	Unknown (FHL1)	HSCT, steroids	Graft failure in HLH	3 mg/kg	21 days	7
3	Merli et al. (2019)	Italy	Prospective study	9 months	F	Familial HLH	UNC13D	HSCT, steroids	Graft failure in HLH	1 mg/kg	18 days	6
4	Alahmari and Khogeer (2023)	Saudi Arabia	Case report	3 years	M	Familial HLH	LYST	DXM, IVIG, tacrolimus, CSA, etoposide	Refractory HLH	50 mg	17 days	6
5	Hahn et al. (2024)	United States	Case report	6 weeks	F	Familial HLH	PRF (homozygous)	DXM, anakinra, IVIG	Severe HLH	6 mg/kg	8 days	3
6	Lam et al. (2019)	Italy	Case report	6 years	F	Familial HLH	NOCARH syndrome	Glucocorticoid, anakinra, CSA	Refractory HLH	NR	NR	NR
7	Schuelke et al. (2023)	United States	Retrospective case series	3.6 months	M	Familial HLH	STXBP2	NR	NR	1 mg/kg	73 days	21
8	Schuelke et al. (2023)	United States	Retrospective case series	2.4 months	M	Familial HLH	STXBP2	NR	NR	1 mg/kg	40 days	13
9	Schuelke et al. (2023)	United States	Retrospective case series	9.6 months	F	Familial HLH	PRF (homozygous)	NR	NR	3 mg/kg	1 day	1
10	Schuelke et al. (2023)	United States	Retrospective case series	4.8 months	M	Familial HLH	PRF (complex heterogeneous)	NR	NR	1 mg/kg	21 days	4
11	Schuelke et al. (2023)	United States	Retrospective case series	13.2 months	M	Familial HLH	PRF (homozygous)	NR	NR	1 mg/kg	116 days	19

No.	Author/Year	Concomitant therapy	Overall response	Time to response	Bridging to HSCT	Post-HSCT outcome	Relapse after emapalumab	Adverse events	Discontinuation due to toxicity	Follow-up duration	Survival/Outcome
1	Merli et al. (2019)	No	Responded	14 days	Yes	Engrafted	No	No	No	24 months	Alive
2	Merli et al. (2019)	No	Responded	13 days	Yes	Engrafted	No	No	No	23 months	Alive
3	Merli et al. (2019)	No	Responded	NR	Yes	Engrafted	No	No	No	21 months	Alive
4	Alahmari and Khogeer (2023)	DXM, tacrolimus	Responded	17 days	Yes	Engrafted	No	Erythematous rash	No	6 months	Alive
5	Hahn et al. (2024)	DXM, anakinra	Responded	6 days	No	NA	No	No	No	21 days	Alive
6	Lam et al. (2019)	Anakinra	Responded	NR	Yes	Engrafted	NR	No	No	6.9 months	Alive
7	Schuelke et al. (2023)	NR	Responded	NR	Yes	Engrafted	No	No	NR	6 months	Alive
8	Schuelke et al. (2023)	NR	Responded	NR	Yes	Engrafted	No	No	NR	6 months	Alive
9	Schuelke et al. (2023)	NR	Responded	NR	Yes	Engrafted	No	No	NR	6 months	Alive
10	Schuelke et al. (2023)	NR	Responded	NR	Yes	Engrafted	No	No	NR	6 months	Alive
11	Schuelke et al. (2023)	NR	Responded	NR	Yes	Engrafted	No	No	NR	6 months	Alive

HLH, hemophagocytic lymphohistiocytosis; HSCT, hematopoietic stem cell transplantation; DXM, dexamethasone; IVIG, intravenous immunoglobulin; CSA, cyclosporine; NR, not reported; NA, not applicable; mg/kg, Milligrams per kilogram; M, male; F, female.

Emapalumab was initiated in critically ill patients with uncontrolled hyperinflammation despite intensive therapy, with the primary aim of achieving rapid disease control and enabling hematopoietic stem-cell transplantation (HSCT). Dosing varied from 1 to 6 mg/kg twice weekly, with one case using a fixed 50 mg regimen (Alahmari and Khogeer, 2023). Treatment duration ranged from 1 day to 4 months (median ∼3 weeks). Concomitant dexamethasone and anakinra were used in several cases.

Clinical outcomes were consistently favorable. All eleven patients (100%) achieved remission or marked improvement within 2 weeks of therapy initiation. Most proceeded to HSCT once inflammation was controlled, achieving successful engraftment without early recurrence. Survival outcomes were excellent: all patients with available follow-up (21 days–24 months) were alive at the last assessment, and no relapses occurred after discontinuation. Only one patient developed a mild erythematous rash (Alahmari and Khogeer, 2023); no other adverse events were reported.

Overall, emapalumab provided rapid and durable suppression of hyperinflammation in infants and children with genetically defined fHLH. As a bridge to HSCT, it achieved complete or partial remission in every case, with minimal drug-related toxicity and sustained survival during medium-term follow-up.

### Emapalumab in rheumatology-associated MAS

Across twelve reported patients from eight publications, emapalumab was used as targeted therapy for MAS secondary to rheumatologic disease (Schuelke et al., 2023; Faggioli et al., 2025; Gabr et al., 2020; Guild et al., 2022; Slaney et al., 2024; Song et al., 2024; Xu et al., 2025; Macaraeg and Schulert, 2023) (Table 2). Cases spanned pediatric and adult populations, ranging from 17 months to 79 years (median ∼18 years). The most common underlying conditions were systemic juvenile idiopathic arthritis (sJIA) and adult-onset Still’s disease (AOSD), accounting for over half of the cases. Other triggers included dermatomyositis, systemic autoimmune arthritis, Sjögren syndrome, and MAS of undetermined rheumatologic etiology. Diagnosis was based on the HLH-2004 criteria (five studies) or the HScore, while others relied on clinical and laboratory features consistent with HLH.

**TABLE 2:** Study characteristics, treatment details, and clinical outcomes of patients with macrophage activation syndrome (MAS) or rheumatology-associated hemophagocytic lymphohistiocytosis (HLH) treated with emapalumab.

No.	Author/Year	Country	Study design	Age	Sex	HLH etiology	Rheumatological disease	Prior treatment before emapalumab	Indication for starting	Emapalumab dose	Duration	Total doses
1	Slaney et al. (2024)	Italy	Case report	2 years	M	Secondary HLH	sJIA	Anakinra, corticosteroids, tacrolimus, CSA	Refractory	1 mg/kg	16 months	128
2	Song et al. (2024)	China	Retrospective	63 years	F	Secondary HLH	SS	NR	Refractory	50 mg	NR	1
3	Song et al. (2024)	China	Retrospective	79 years	M	Secondary HLH	AOSD	NR	Refractory	50 mg	NR	4
4	Xu et al. (2025)	China	Retrospective	54 years	M	Secondary HLH	AOSD	Glucocorticoids, IVIG, methotrexate, baricitinib	Refractory	NR	NR	NR
5	Xu et al. (2025)	China	Retrospective	58 years	F	Secondary HLH	AOSD	Glucocorticoids, CSA, IVIG, baricitinib	Refractory	NR	NR	NR
6	Faggioli et al. (2025)	Italy	Case report	41 years	F	Secondary HLH	Dermatomyositis	MP, anakinra, eculizumab	Refractory	6 mg/kg	28 days	3–9
7	Gabr et al. (2020)	United States	Case report	22 years	F	Secondary HLH	AOSD	MP, anakinra	Refractory	1 mg/kg	44 days	10
8	Guild et al. (2022)	United States	Case report	17 months	M	Secondary HLH	sJIA	Anakinra, prednisolone, canakinumab, MP	Recurrent	1 mg/kg	10 weeks	20
9	Macaraeg and Schulert (2023)	United States	Case report	20 years	M	Secondary HLH	sJIA	Intravenous MP	Refractory	NR	19 days	6
10	Schuelke et al. (2023)	United States	Retrospective	15 years	F	Secondary HLH	MAS (unknown primary disease)	NR	NR	1 mg/kg	1 day	1
11	Schuelke et al. (2023)	United States	Retrospective	11 years	F	Secondary HLH	sJIA	NR	NR	1 mg/kg	15 days	3
12	Schuelke et al. (2023)	United States	Retrospective	12 years	F	Secondary HLH	sJIA	NR	NR	1 mg/kg	17 days	6

No.	Author/Year	Concomitant therapy	Overall response	Time to response	Bridging to HSCT	Post-HSCT outcome	Relapse after emapalumab	Adverse events	Discontinuation due to toxicity	Follow-up duration	Survival/Outcome
1	Slaney et al. (2024)	Anakinra, corticosteroids, tacrolimus, CSA, etoposide, IVIG	Responded	NR	No	NA	No	No	No	24 months	Alive
2	Song et al. (2024)	Ruxolitinib	Responded	21.1 weeks	No	NA	No	No	No	5.8 months (median)	Alive
3	Song et al. (2024)	Ruxolitinib, VP-16, MP	No response	3.9 weeks	No	NA	NA	NA	No	5.8 months (median)	Dead
4	Xu et al. (2025)	No	Responded	NR	No	NA	No	NR	NR	12 months	Alive
5	Xu et al. (2025)	No	Responded	NR	No	NA	NR	NR	NR	12 months	Alive
6	Faggioli et al. (2025)	No	Responded	NR	No	No	No	No	No	9 months	Alive
7	Gabr et al. (2020)	DXM	Responded	1 day	No	NA	No	No	No	36 days	Alive
8	Guild et al. (2022)	Anakinra, DXM	Responded	NR	No	NA	No	No	No	3.5 years	Alive
9	Macaraeg and Schulert (2023)	Canakinumab, prednisolone	Responded	NR	No	NA	Yes	No	No	40 days	Alive
10	Schuelke et al. (2023)	NR	Responded	NR	No	NA	No	No	No	6 months	Alive
11	Schuelke et al. (2023)	NR	Responded	NR	No	NA	No	No	No	6 months	Alive
12	Schuelke et al. (2023)	NR	Responded	NR	No	NA	No	No	No	6 months	Alive

HLH, hemophagocytic lymphohistiocytosis; MAS, macrophage activation syndrome; HSCT, hematopoietic stem cell transplantation; sJIA, systemic juvenile idiopathic arthritis; AOSD, Adult-Onset Still’s Disease; SS, Sjögren Syndrome; MP, methylprednisolone; DXM, dexamethasone; CSA, cyclosporine; IVIG, intravenous immunoglobulin; VP-16, Etoposide; NR, not reported; NA, not applicable; mg/kg, Milligrams per kilogram; M, male; F, female.

Emapalumab was administered in severe or refractory MAS unresponsive to high-dose corticosteroids and other immunomodulators. Nearly all patients received glucocorticoids; several had also been treated with anakinra, cyclosporine, tacrolimus, tocilizumab, etoposide, or IVIg. The typical indication for emapalumab was persistent MAS despite combination immunosuppression or cytokine blockade. Dosing schedules varied, usually involving multiple infusions over 2–3 weeks, with total treatment durations ranging from 1 day to 16 months. One patient received 128 doses over 16 months (Slaney et al., 2024), whereas another responded after a single dose (Schuelke et al., 2023). Concomitant corticosteroids were nearly universal; some patients also received anakinra, IVIg, or ruxolitinib. None received emapalumab as a bridge to HSCT.

Clinical outcomes were largely favorable. Clinical improvement was observed in 11 of 12 patients (91.7%), with a median response time of approximately 12 days. Most maintained sustained remission during follow-up (2–24 months). One patient (Song et al., 2024) did not respond and died. No relapses occurred after discontinuation, and the drug was well tolerated with no reported toxicity.

Collectively, real-world data support emapalumab as an effective and well-tolerated cytokine-directed therapy for refractory rheumatology-associated MAS, leading to rapid stabilization and sustained remission.

### Emapalumab in malignancy-associated HLH

A total of twenty-five patients across six published reports received emapalumab for HLH secondary to malignancy (Johnson et al., 2024; Schuelke et al., 2023; Song et al., 2024; Krishnareddigari et al., 2025; Maher et al., 2025; Goldstein et al., 2023) (Table 3). The underlying malignancies were exclusively hematologic, dominated by T- and NK-cell lymphomas, including peripheral T-cell lymphoma not otherwise specified, angioimmunoblastic T-cell lymphoma, NK/T-cell lymphoma, HTLV-1–associated adult T-cell leukemia/lymphoma, EBV-positive diffuse large B-cell lymphoma, and myelodysplastic syndrome with excess blasts (MDS-EB2). One case involved Waldenström macroglobulinemia. The affected population consisted primarily of adolescents and adults between 16 and 73 years of age, with a slight male predominance. In nearly all cases, diagnostic confirmation was based on the HLH-2004 criteria.

**TABLE 3:** Study characteristics, treatment details, and clinical outcomes of patients with malignancy-associated hemophagocytic lymphohistiocytosis (HLH) treated with emapalumab.

No.	Author/Year	Country	Study design	Age	Sex	HLH etiology	Type of malignancy	Prior treatment before emapalumab	Indication	Emapalumab dose	Treatment duration	Total doses
1	Maher et al. (2025)	United States	Case report	17 years	M	Secondary HLH	SEBVTCL (childhood)	Anakinra, steroids, IVIG, rituximab	Refractory	NR	NR	NR
2	Song et al. (2024)	China	Retrospective	18 years	M	Secondary HLH	Lymphoma	HLH-94/04, steroids, IVIG	Refractory	50 mg	1.14 weeks (median)	1
3	Song et al. (2024)	China	Retrospective	54 years	M	Secondary HLH	PTCL	HLH-94/04, steroids, IVIG	Refractory	50 mg	1.14 weeks	2
4	Song et al. (2024)	China	Retrospective	34 years	M	Secondary HLH	Lymphoma	HLH-94/04, steroids, IVIG	Refractory	100 mg	1.14 weeks	1
5	Song et al. (2024)	China	Retrospective	38 years	F	Secondary HLH	Lymphoma	HLH-94/04, steroids, IVIG	Refractory	50 mg	1.14 weeks	1
6	Song et al. (2024)	China	Retrospective	33 years	M	Secondary HLH	NK/T-cell lymphoma	HLH-94/04, steroids, IVIG	Refractory	50 mg	1.14 weeks	2
7	Krishnareddigari et al. (2025)	United States	Case report	16 years	F	Secondary HLH	NK/T-cell lymphoma (nasopharynx)	DXM, etoposide, rituximab	Refractory	NR	NR	NR
8	Krishnareddigari et al. (2025)	United States	Case report	17 years	M	Secondary HLH	T-ALL	DXM, IVIG, hydrocortisone, ruxolitinib, etoposide	Refractory	NR	3 months	NR
9	Schuelke et al. (2023)	United States	Retrospective	16 years	F	Secondary HLH	NK lymphoma/EBV viremia	NR	Refractory	3 mg/kg	1 day	1
10	Johnson et al. (2024)	United States	Retrospective	65 years	NR	Secondary HLH	EBV + DLBCL/MF IVB	Steroids, etoposide	Refractory	1 mg/kg	5 days	1
11	Johnson et al. (2024)	United States	Retrospective	58 years	NR	Secondary HLH	PTCL-NOS	Steroids, etoposide	Refractory	1 mg/kg	1 day	1
12	Johnson et al. (2024)	United States	Retrospective	73 years	NR	Secondary HLH	PTCL-NOS	Steroids, etoposide	Refractory	1 mg/kg	13 days	4
13	Johnson et al. (2024)	United States	Retrospective	69 years	NR	Secondary HLH	THRLBCL	Steroids, etoposide	Refractory	1 mg/kg	5 days	1
14	Johnson et al. (2024)	United States	Retrospective	54 years	NR	Secondary HLH	EBV + PTLD	Steroids, etoposide	Refractory	1 mg/kg	10 days	3
15	Johnson et al. (2024)	United States	Retrospective	46 years	NR	Secondary HLH	ALK-ALCL	Steroids, etoposide	Refractory	1 mg/kg	5 days	1
16	Johnson et al. (2024)	United States	Retrospective	54 years	NR	Secondary HLH	PTCL-TFH	Steroids, etoposide	Refractory	1 mg/kg	18 days	2
17	Johnson et al. (2024)	United States	Retrospective	69 years	NR	Secondary HLH	PTCL-NOS	Steroids, etoposide	Refractory	1 mg/kg	15 days	4
18	Johnson et al. (2024)	United States	Retrospective	61 years	NR	Secondary HLH	EBV + DLBCL	Steroids, etoposide	Refractory	1 mg/kg	1 day	1
19	Johnson et al. (2024)	United States	Retrospective	67 years	NR	Secondary HLH	AITL	Steroids, etoposide	Refractory	1 mg/kg	23 days	4
20	Johnson et al. (2024)	United States	Retrospective	69 years	NR	Secondary HLH	PTCL-NOS	Steroids, etoposide	Refractory	1 mg/kg	13 days	4
21	Johnson et al. (2024)	United States	Retrospective	65 years	NR	Secondary HLH	WM	Steroids, etoposide	Refractory	1 mg/kg	3 days	1
22	Johnson et al. (2024)	United States	Retrospective	71 years	F	Secondary HLH	MDS-EB2	Steroids, etoposide	Refractory	1 mg/kg	40 days	7
23	Johnson et al. (2024)	United States	Retrospective	69 years	NR	Secondary HLH	HTLV-1+ ATLL	Steroids, etoposide	Refractory	1 mg/kg	4 days	1
24	Johnson et al. (2024)	United States	Retrospective	53 years	NR	Secondary HLH	ENKTL	Steroids, etoposide	Refractory	1 mg/kg	5 days	2
25	Goldstein et al. (2023)	United States	Case report	22 mth	NR	Secondary HLH	SEBVTCL (childhood)	DXM	Refractory	NR	2 days	NR

No.	Author/Year	Concomitant therapy	Overall response	Time to response	Bridging to HSCT	Post-HSCT outcome	Relapse	Adverse events	Discontinuation due to toxicity	Follow-up duration	Survival/Outcome
1	Maher et al. (2025)	Anakinra, steroids, IVIG, rituximab	NR	NR	No	NA	NA	No	NA	13 days	NR/DAMA
2	Song et al. (2024)	Ruxolitinib, DEP	No response	NA	No	NA	NA	No	No	5.8 months (median)	Dead
3	Song et al. (2024)	Ruxolitinib, DEP, Hyper-CVAD A	No response	2.8 weeks	No	NA	NA	No	No	5.8 months (median)	Dead
4	Song et al. (2024)	Ruxolitinib, VP-16, Hyper-CVAD A	Responded	1.7 weeks	Yes	Engrafted	No	NR	NR	5.8 months (median)	Alive
5	Song et al. (2024)	Ruxolitinib, venetoclax	Responded	29.8 weeks	No	No	No	No	No	5.8 months (median)	Alive
6	Song et al. (2024)	DEP + rituximab, PD-1 blockade	Responded	NR	No	No	No	No	No	5.8 months (median)	Alive
7	Krishnareddigari et al. (2025)	No	No response	NA	No	NA	NA	Fulminant candidiasis, ICH	No	NR	Dead
8	Krishnareddigari et al. (2025)	No	Responded	7 days	No	No	No	No	No	NR	Alive
9	Schuelke et al. (2023)	NR	No response	NA	No	No	NA	NR	No	6 months	Dead
10	Johnson et al. (2024)	Steroids, etoposide	No response	NA	No	NA	NA	No	No	4 days	Dead
11	Johnson et al. (2024)	Steroids, etoposide	No response	NA	No	NA	NA	No	No	1 day	Dead
12	Johnson et al. (2024)	Steroids, etoposide	No response	NA	No	NA	NA	No	No	13 days	Dead
13	Johnson et al. (2024)	Steroids, etoposide	No response	NA	No	NA	NA	No	No	5 days	Dead
14	Johnson et al. (2024)	Steroids, etoposide	No response	NA	No	NA	NA	No	No	10 days	Dead
15	Johnson et al. (2024)	Steroids, etoposide	No response	NA	No	NA	NA	No	No	5 days	Dead
16	Johnson et al. (2024)	Steroids, etoposide	No response	NA	No	NA	NA	No	No	18 days	Dead
17	Johnson et al. (2024)	Steroids, etoposide	No response	NA	No	NA	NA	No	No	15 days	Dead
18	Johnson et al. (2024)	Steroids, etoposide	No response	NA	No	NA	NA	No	No	1 day	Dead
19	Johnson et al. (2024)	Steroids, etoposide	No response	NA	No	NA	NA	No	No	23 days	Dead
20	Johnson et al. (2024)	Steroids, etoposide	No response	NA	No	NA	NA	No	No	13 days	Dead
21	Johnson et al. (2024)	Steroids, etoposide	No response	NA	No	NA	NA	No	No	3 days	Dead
22	Johnson et al. (2024)	Steroids, etoposide	Responded	20 days	Yes	NR	No	No	No	257 days	Alive
23	Johnson et al. (2024)	Steroids, etoposide	No response	NA	No	NA	No	No	No	4 days	Dead
24	Johnson et al. (2024)	Steroids, etoposide	No response	NA	No	NA	No	No	No	5 days	Dead
25	Goldstein et al. (2023)	DXM, anakinra, eculizumab	Responded	4 days	Yes	Engrafted	No	No	No	8 months	Alive

HLH, hemophagocytic lymphohistiocytosis; HSCT, hematopoietic stem cell transplantation; IVIG, intravenous immunoglobulin; DXM, dexamethasone; MP, methylprednisolone; VP-16, Etoposide; NR, not reported; NA, not applicable; DAMA, discharge against medical advice; mg/kg, Milligrams per kilogram; yrs, years; mth, months.

Emapalumab was administered as rescue therapy for uncontrolled hyperinflammation unresponsive to conventional regimens such as HLH-94 or HLH-2004 protocols and other cytokine-directed agents. Malignancy-directed therapy was reported in 15 of 25 patients with malignancy-associated HLH. Nearly all patients had received high-dose corticosteroids before starting emapalumab, and most had been exposed to etoposide or ruxolitinib. Several also received intravenous immunoglobulin, tocilizumab, or calcineurin inhibitors, anakinra, and rituximab. The decision to initiate emapalumab was typically driven by persistent cytopenias, progressive organ dysfunction, or refractory hyperferritinemia despite prior therapy. Dosing approaches varied across studies, with weight-based administration (1–10 mg/kg) being most common and fixed-dose 50 mg regimens used in a minority of cases. The duration of therapy was generally short, with a median of approximately 5 days, although some patients continued treatment for weeks to months. Three patients received emapalumab as a bridge to hematopoietic stem-cell transplantation.

Clinical responses to emapalumab were observed in a subset of patients, although outcomes were highly dependent on the severity of the underlying malignancy and organ failure at the time of treatment. Only six of twenty-five patients (24%) demonstrated clinical improvement, underscoring the inferior outcomes in malignancy-associated HLH compared with other subtypes. The median time to clinical improvement among responders was approximately 1 week. Response rates differed markedly between reports. In the Johnson et al. (2024) series of fifteen adults with advanced or relapsed malignancies, only one patient achieved a partial response, and the remaining fourteen died despite treatment. In contrast, among the ten patients reported outside the Johnson et al. (2024) cohort, five demonstrated clinical improvement and four failed to respond. These patients tended to have less extensive disease and were treated earlier in their HLH course. Sustained remission following discontinuation of emapalumab was documented in several cases, with follow-up extending to 8 months.

Drug-related toxicity was infrequent. The most serious adverse outcome was a single case of disseminated candidiasis with intracranial hemorrhage reported by Krishnareddigari et al. (2025). No patients discontinued emapalumab due to toxicity. The remaining complications were attributed to preexisting organ dysfunction, malignant progression, or infection arising from severe immunosuppression. The median time to response across studies was approximately 7 days, consistent with the pharmacodynamic onset of interferon-γ neutralization observed in prior pediatric HLH data.

Overall survival in this collective experience remained poor, reflecting the aggressive nature of the underlying malignancies rather than direct effects of emapalumab. At last follow-up, six patients were alive and eighteen had died. Within the Johnson et al. (2024) cohort, which included heavily pretreated adults with refractory lymphoma or myelodysplastic disease, fourteen of fifteen patients died within weeks of HLH diagnosis, and the median overall survival was 18 days from diagnosis and 4 days from initiation of emapalumab. In contrast, survival was more favorable in non-Johnson et al. (2024) reports, in which five of ten patients remained alive at follow-up periods extending beyond several months. These included patients with T-cell lymphomas who responded clinically to emapalumab, some of whom proceeded successfully to transplantation.

Taken together, these data indicate that emapalumab was predominantly used as a late-line, short-course rescue therapy in patients with severe, refractory malignancy-associated HLH. While individual cases demonstrated clinical benefit and short-term control of hyperinflammation, overall outcomes were largely determined by the progression of the underlying malignancy and the extent of multiorgan failure at treatment initiation. The available evidence suggests that emapalumab can achieve meaningful anti-inflammatory responses in selected patients but should be considered primarily as a component of multimodal salvage therapy or as a bridge to curative intervention such as hematopoietic stem-cell transplantation, rather than as a stand-alone curative approach in advanced M-HLH.

### Emapalumab in infection-associated HLH

A total of twenty-two patients from eleven published reports received emapalumab for infection-associated secondary HLH (Wang et al., 2024; Schuelke et al., 2023; Song et al., 2024; Dupuy et al., 2022; Jia et al., 2024; Lounder et al., 2019; McKeone et al., 2021; Squire et al., 2020; Triebwasser et al., 2021; Zhu et al., 2025; Tucci et al., 2021) (Table 4). All cases represented secondary HLH triggered by infectious agents rather than primary or familial disease. The underlying infections were predominantly viral, with Epstein–Barr virus identified as the causative trigger in nineteen patients, including multiple cases of chronic active EBV infection. Other infectious etiologies included disseminated HSV-1, *Burkholderia multivorans* sepsis, BCGitis with concurrent adenoviral and fungal coinfection, and EBV-associated T-cell lymphoma. Patients ranged in age from early childhood to middle adulthood, encompassing both pediatric and adult cases. Diagnostic confirmation followed the HLH-2004 criteria in fifteen of the twenty-two patients.

**TABLE 4:** Study characteristics, treatment details, and clinical outcomes of patients with infection-associated secondary hemophagocytic lymphohistiocytosis (HLH) treated with emapalumab.

No.	Author/Year	Country	Study design	Age	Sex	HLH etiology	Type of infection	Prior treatment before emapalumab	Indication	Emapalumab dose	Duration	Total doses
1	McKeone et al. (2022)	United States	Case report	11 days	F	Secondary HLH	HSV-1	DXM, etoposide, IVIG, anakinra	Refractory	6 mg/kg	1 day	1
2	Song et al. (2024)	China	Retrospective case series	25 years	M	Secondary HLH	EBV	HLH-94/04	Refractory	50 mg	1.14 weeks (median)	2
3	Song et al. (2024)	China	Retrospective case series	24 years	M	Secondary HLH	EBV	HLH-94/04	Refractory	50 mg	1.14 weeks (median)	1
4	Song et al. (2024)	China	Retrospective case series	46 years	M	Secondary HLH	EBV	HLH-94/04	Refractory	50 mg	1.14 weeks (median)	1
5	Song et al. (2024)	China	Retrospective case series	33 years	M	Secondary HLH	EBV	HLH-94/04	Refractory	50 mg	1.14 weeks (median)	1
6	Song et al. (2024)	China	Retrospective case series	29 years	M	Secondary HLH	EBV	HLH-94/04	Refractory	100 mg	1.14 weeks (median)	2
7	Zhu et al. (2025)	China	Retrospective case series	29 years	M	Secondary HLH	EBV	Ruxolitinib, MP, PD-1 mAb	Refractory	1 mg/kg	5 days	2
8	Zhu et al. (2025)	China	Retrospective case series	33 years	M	Secondary HLH	EBV	PD-1 mAb, ruxolitinib + CD20 mAb	Refractory	1 mg/kg	4 days	2
9	Zhu et al. (2025)	China	Retrospective case series	25 years	M	Secondary HLH	EBV	DXM + IVIG	Refractory	1 mg/kg	1 day	1
10	Wang et al. (2024)	China	Case series	6 years	F	Secondary HLH	EBV	Steroids, etoposide, ruxolitinib, IVIG	Refractory	1 mg/kg	1 day	4
11	Wang et al. (2024)	China	Case series	2 years	M	Secondary HLH	EBV	L-DEP protocol	Refractory	NR	1 day	1
12	Wang et al. (2024)	China	Case series	8 years	F	Secondary HLH	EBV	HLH-94 protocol	Refractory	NR	1 day	1
13	Wang et al. (2024)	China	Case series	10 years	M	Secondary HLH	EBV	HLH-94 protocol	Refractory	NR	1 day	1
14	Triebwasser et al. (2021)	United States	Case report	26 years	M	Secondary HLH	CA-EBV	HLH-2004, CSA, anakinra	Refractory	NR	2 months	17
15	Squire et al. (2020)	United States	Case report	17 years	M	Secondary HLH	*Burkholderia multivorans* sepsis	IVIG, corticosteroids, basiliximab	Refractory	NR	NR	1
16	Tucci et al. (2021)	Italy	Case report	4 years	F	Secondary HLH	Disseminated BCGitis + adenovirus + *Stenotrophomonas* + pulmonary aspergillosis	MP, IVIG	Refractory	1 mg/kg	8 weeks	15
17	Schuelke et al. (2023)	United States	Retrospective	26 years	M	Secondary HLH	CA-EBV	NR	NR	1 mg/kg	51 days	15
18	Schuelke et al. (2023)	United States	Retrospective	27 years	M	Secondary HLH	CAEBV/PTCL	NR	NR	1 mg/kg	12 days	3
19	Schuelke et al. (2023)	United States	Retrospective	12 years	F	Secondary HLH	EBV viremia	NR	NR	1 mg/kg	4 days	2
20	Lounder et al. (2019)	United States	Case report	20 mth	M	Secondary HLH	EBV	HLH-94, rituximab, IVIG	Refractory	NR	14 weeks	NR
21	Dupuy et al. (2020)	United States	Case report	14 years	M	Secondary HLH	EBV	Anakinra, DXM, IVIG, tofacitinib	Refractory	NR	NR	NR
22	Jia et al. (2024)	China	Case report	5 years	F	Secondary HLH	EBV	HLH-94, ruxolitinib, L-DEP	Relapse/Refractory	1 mg/kg	6 days	3

No.	Author/Year	Concomitant therapy	Overall response	Time to response	Bridging to HSCT	Post-HSCT outcome	Relapse	Adverse events	Discontinuation due to toxicity	Follow-up duration	Survival/Outcome
1	McKeone et al. (2022)	Anakinra, IVIG	No response	NA	No	NA	NA	No	No	6 days	Dead
2	Song et al. (2024)	Ruxolitinib, MP, PD-1 blockade, DEP	Responded	11.3 weeks	Yes	Engrafted	No	No	No	5.8 months (median)	Alive
3	Song et al. (2024)	Ruxolitinib, MP, PD-1 blockade	Responded	10.4 weeks	Yes	Engrafted	No	No	No	5.8 months (median)	Alive
4	Song et al. (2024)	Ruxolitinib, PD-1 blockade	No response	NA	NA	NA	No	NA	No	NR	Dead
5	Song et al. (2024)	PD-1 blockade	Responded	1 week	Yes	Engrafted	No	No	No	5.8 months (median)	Alive
6	Song et al. (2024)	Ruxolitinib, DXM, IVIG, PD-1 blockade	Responded	18.9 weeks	Yes	Engrafted	NR	No	NR	5.8 months (median)	Alive
7	Zhu et al. (2025)	Ruxolitinib, etoposide, MP, PD-1 mAb	Responded	1 day	Yes	Engrafted	No	No	No	3 months	Alive
8	Zhu et al. (2025)	PD-1 mAb, ruxolitinib, CD20 mAb, oral etoposide + DEP	Responded	1 day	Yes	Engrafted	No	No	No	5 months	Alive
9	Zhu et al. (2025)	Ruxolitinib, MP, PD-1 mAb, DEP	Responded	1 day	Yes	Engrafted	No	No	No	3 months	Alive
10	Wang et al. (2024)	Etoposide, ruxolitinib, DXM	No response	NA	No	NA	NA	Epistaxis, intracranial hypertension	No	6 days	Dead
11	Wang et al. (2024)	Second L-DEP cycle	Responded	1 day	Yes	Engrafted	No	NR	No	NR	Alive
12	Wang et al. (2024)	No	Responded	1 day	No	NR	No	No	No	22 days	Alive
13	Wang et al. (2024)	Etoposide, DXM, ruxolitinib	Responded	1 day	No	NA	No	No	No	15 days	Alive
14	Triebwasser et al. (2021)	Ruxolitinib	Responded	NR	Yes	Engrafted	NR	HSV stomatitis and viremia	No	5 months	Alive
15	Squire et al. (2020)	Nil	No response	NA	No	NA	NA	NR	NR	33 days	Dead
16	Tucci et al. (2021)	MP, CSA	Responded	NR	Yes	Engrafted	No	No	No	9 months	Alive
17	Schuelke et al. (2023)	NR	Responded	NR	Yes	Engrafted	Yes	No	No	6 months	Alive
18	Schuelke et al. (2023)	NR	Responded	NR	Yes	Engrafted	NR	NR	NR	6 months	Alive
19	Schuelke et al. (2023)	NR	No response	NR	NR	No	NR	NR	NR	6 months	Dead
20	Lounder et al. (2019)	DXM	Responded	1 day	No	NA	No	No	No	36 months	Alive
21	Dupuy et al. (2020)	Anakinra, DXM, IVIG, tofacitinib	No response	NA	No	NA	NA	No	No	Days	Dead
22	Jia et al. (2024)	DXM	Responded	6 days	Yes	Engrafted	No	No	No	15 months	Alive

HLH, hemophagocytic lymphohistiocytosis; HSCT, hematopoietic stem cell transplantation; EBV, Epstein–Barr Virus; HSV-1, Herpes Simplex Virus type 1; CA-EBV, Chronic Active Epstein–Barr Virus; PTCL, Peripheral T-cell Lymphoma; CSA, cyclosporine; DEP, Doxorubicin–Etoposide–Prednisolone; L-DEP, Liposomal Doxorubicin–Etoposide–Prednisolone; DXM, dexamethasone; MP, methylprednisolone; PD-1, Programmed Cell Death Protein 1; PD-1, mAb, Programmed Cell Death Protein one monoclonal antibody; IVIG, intravenous immunoglobulin; NR, not reported; NA, not applicable; mg/kg, Milligrams per kilogram; yrs, years; mth, months; wks, weeks.

Emapalumab was initiated after failure of standard HLH-directed therapy, most often high-dose corticosteroids with or without etoposide. Several patients had also received or were concurrently treated with ruxolitinib, cyclosporine, tacrolimus, anakinra, or intravenous immunoglobulin. The rationale for initiating interferon-γ blockade was persistent or relapsing hyperinflammation, manifested by fever, cytopenias, and rising ferritin levels despite conventional therapy. Treatment regimens were primarily weight-based, typically ranging from 1 to 6 mg/kg per dose. Fixed-dose strategies were not prominent in this infection-associated cohort. The median number of administered doses was two, and the median treatment duration was approximately two and a half days, although individual courses extended up to several weeks in selected reports. The median time to clinical improvement was approximately 1 day, consistent with rapid biochemical and hematologic recovery once interferon-γ signaling was suppressed. Corticosteroids were universally co-administered, while ruxolitinib and IVIg were the most frequent adjuncts.

Emapalumab induced clinical remission in 16 of 22 patients (72.7%), with most achieving improvement within 1 day of therapy. Emapalumab was frequently used as a bridge to hematopoietic stem-cell transplantation, with thirteen patients proceeding to transplant following disease control. All transplanted individuals achieved engraftment, and short-term survival post-transplant was reported as favorable across cases. Among the six nonresponders, mortality was most often linked to uncontrolled infection or progression of EBV-associated lymphoproliferative disease rather than emapalumab failure. Overall, sixteen patients were alive at last follow-up, and six had died. The observed deaths were attributed to infection or malignancy-related complications rather than drug toxicity.

Reported toxicities included transient epistaxis, intracranial hypertension, and herpetic stomatitis with viremia; none necessitated treatment discontinuation. No severe or fatal drug-related reactions were documented. The overall safety profile was favorable, and emapalumab was well tolerated even in critically ill patients with ongoing infectious processes.

In summary, across twenty-two infection-associated HLH cases from eleven reports, emapalumab achieved rapid and durable suppression of hyperinflammation in approximately three-quarters of treated patients. It successfully facilitated hematopoietic stem-cell transplantation in more than half, with excellent short-term engraftment outcomes and minimal drug-related toxicity. Although long-term prognosis remained largely dependent on infection control and the success of subsequent transplantation, the collective evidence from these studies demonstrates that interferon-γ blockade with emapalumab provides an effective and well-tolerated therapeutic option for refractory infection-associated HLH, particularly in EBV-driven disease.

### Emapalumab in refractory CRS/IEC-HS

Across six publications, eleven patients developed HLH secondary to CAR-T–related toxicity or severe cytokine release syndrome (CRS) and were treated with emapalumab (Schuelke et al., 2023; McNerney et al., 2022; Rainone et al., 2023; Manghisi et al., 2025; Ruemmele et al., 2025; Scala et al., 2024) (Table 5). Ages ranged from 5 to 67 years (median 19). Diagnostic methods varied: HLH-2004 criteria and HScore were each used in one case; others relied on compatible clinical and laboratory findings.

**TABLE 5:** Study characteristics, treatment details, and clinical outcomes of patients with CAR-T or immune effector cell–associated HLH-like syndrome (IEC-HS) treated with emapalumab.

No.	Author/Year	Country	Study design	Age	Sex	HLH etiology	Prior treatment before emapalumab	Indication	Emapalumab dose	Duration	Total doses
1	Manghisi et al. (2025)	Italy	Case report	30 years	M	CAR-T–related HLH (IEC-HS)	Alemtuzumab, ruxolitinib, tocilizumab, DXM, anakinra, MP, siltuximab	Refractory IEC-HS	1 mg/kg	14 days	4
2	Schuelke et al. (2023)	United States	Retrospective	16 years	M	CAR-T–related HLH	Tocilizumab, DXM, anakinra	Refractory CRS	1 mg/kg	1 day	1
3	Schuelke et al. (2023)	United States	Retrospective	5 years	F	CAR-T–related HLH	Tocilizumab, MP	Refractory CRS	1 mg/kg	1 day	1
4	Schuelke et al. (2023)	United States	Retrospective	5 years	M	CAR-T–related HLH	Tocilizumab, MP, anakinra	Refractory CRS	1 mg/kg	1 day	1
5	Schuelke et al. (2023)	United States	Retrospective	19 years	M	CAR-T–related HLH	Tocilizumab, methylprednisolone	Refractory CRS	1 mg/kg	1 day	1
6	Schuelke et al. (2023)	United States	Retrospective	12 years	F	CAR-T–related HLH	Tocilizumab, MP, siltuximab	Refractory CRS	1 mg/kg	1 day	1
7	Schuelke et al. (2023)	United States	Retrospective	20 years	M	CAR-T–related HLH	Tocilizumab, DXM, anakinra	Refractory CRS	1 mg/kg	1 day	1
8	McNerney et al. (2022)	United States	Case report	19 years	M	CAR-T–related HLH	Tocilizumab, corticosteroids, siltuximab	Refractory CRS	1 mg/kg	1 day	1
9	Ruemmele et al. (2025)	United States	Case report	67 years	M	CAR-T–related HLH	Siltuximab, MP, anakinra, tocilizumab, ruxolitinib, DXM	Refractory CRS	1 mg/kg	4 days	2
10	Rainone et al. (2023)	United States	Case report	19 years	M	CAR-T–related HLH	Tocilizumab, DXM, anakinra, venetoclax	Refractory CRS	1 mg/kg	9 days	4
11	Scala et al. (2024)	United States	Case series	16 years	M	CAR-T–related HLH (IEC-HS)	Anakinra, DXM	Refractory IEC-HS	100 mg	1 day	1

No.	Author/Year	Concomitant therapy	Overall response	Time to response	Bridging to HSCT	Post-HSCT outcome	Relapse	Adverse events	Discontinuation due to toxicity	Follow-up duration	Survival/Outcome
1	Manghisi et al. (2025)	Corticosteroids, anakinra, ruxolitinib	Responded	72 h	Yes	Engrafted	Yes	GI bleed	No	3 months	Dead
2	Schuelke et al. (2023)	DXM, anakinra	Responded	2 days	No	NA	NR	No	No	6 months	Alive
3	Schuelke et al. (2023)	Anakinra	Responded	1 day	No	NA	NR	No	No	6 months	Alive
4	Schuelke et al. (2023)	Anakinra, steroids	No response	1 day	NA	NA	NR	Cerebral edema	No	NA	Dead
5	Schuelke et al. (2023)	NR	Responded	5 days	No	NA	NR	AKI	No	6 months	Alive
6	Schuelke et al. (2023)	Siltuximab, vasoactive agents	Responded	1 day	No	NA	NR	No	NR	6 months	Alive
7	Schuelke et al. (2023)	Siltuximab	Responded	2 days	No	NA	NR	*Enterococcus faecium* bacteremia	No	6 months	Alive
8	McNerney et al. (2022)	Corticosteroids	Responded	1 day	No	NA	No	No	No	12 months	Alive
9	Ruemmele et al. (2025)	NR	Responded	12 h	NA	NA	NR	No	NR	37 days	Dead
10	Rainone et al. (2023)	DXM, anakinra	Responded	12 h	No	NA	NR	No	No	29 days	Alive
11	Scala et al. (2024)	DXM, anakinra	Responded	3 h	No	NA	No	No	No	65 days	Alive

HLH, hemophagocytic lymphohistiocytosis; CAR-T, Chimeric Antigen Receptor T-Cell; IEC-HS, Immune Effector Cell–Associated HLH-like Syndrome; CRS, cytokine release syndrome; HSCT, hematopoietic stem cell transplantation; DXM, dexamethasone; MP, methylprednisolone; NR, not reported; NA, not applicable; AKI, acute kidney injury; GI, gastrointestinal; mg/kg, Milligrams per kilogram; h, hours; yrs, years.

Emapalumab was introduced for persistent hyperinflammation despite standard interventions, including tocilizumab, dexamethasone, anakinra, and ruxolitinib. Reported treatment duration ranged from one to 14 days (median one), with one to four doses (median one). One patient received emapalumab as a bridge to HSCT (Manghisi et al., 2025).

Ten of eleven patients (90.9%) achieved rapid suppression of hyperinflammation after one or a few doses. At last follow-up, eight patients were alive and three had died (follow-up 1–12 months). Reported adverse events were unrelated to emapalumab, including transient gastrointestinal bleeding (Manghisi et al., 2025), cerebral edema due to cytokine storm (Schuelke et al., 2023), acute kidney injury (Ruemmele et al., 2025), and *Enterococcus faecium* bacteremia (Rainone et al., 2023). No treatment discontinuations occurred.

Overall, emapalumab produced rapid and sustained suppression of hyperinflammation in CAR-T/CRS-associated HLH, with most patients stabilizing after one or a few doses and minimal drug-related toxicity.

### Emapalumab in other secondary HLH

Across five reported cases from four publications, emapalumab was administered for secondary hemophagocytic lymphohistiocytosis not attributable to infection, malignancy, rheumatologic disease, or CAR-T therapy (Schuelke et al., 2023; Guild et al., 2022; Song et al., 2024; Wu et al., 2022) (Table 6). All cases were non-familial and occurred in young to middle-aged adults. Diagnostic confirmation varied across reports: two studies used the HLH-2004 criteria, one applied the HScore, and two relied on compatible clinical and laboratory findings indicative of HLH.

**TABLE 6:** Study characteristics, treatment details, and clinical outcomes of patients with other secondary or idiopathic hemophagocytic lymphohistiocytosis (HLH) treated with emapalumab.

No.	Author/Year	Country	Study design	Age	Sex	HLH etiology	Specification	Prior treatment before emapalumab	Indication	Emapalumab dose	Duration	Total doses
1	Song et al. (2024)	China	Retrospective case series	35 years	F	Secondary HLH	Unknown etiology	NR	Refractory	50 mg	1.14 weeks	2
2	Wu et al. (2022)	United States	Prospective study	32 years	F	Secondary HLH	Post–COVID-19 mRNA vaccine	Prednisone, etoposide, DXM	Refractory HLH	NR	NR	8
3	Guild et al. (2022)	United States	Retrospective case series	3 years	F	Secondary HLH	HLH in trisomy 21	Anakinra, DXM, etoposide	Refractory	1 mg/kg	10 weeks	20
4	Schuelke et al. (2023)	United States	Retrospective	0.5 years	M	Secondary HLH	Severe aplastic anemia	NR	NR	1 mg/kg	19 days	7
5	Schuelke et al. (2023)	United States	Retrospective	9 years	M	Secondary HLH	Severe aplastic anemia	NR	NR	1 mg/kg	14 days	3

No.	Author/Year	Concomitant therapy	Overall response	Time to response	Bridging to HSCT	Post-HSCT outcome	Relapse	Adverse events	Discontinuation due to toxicity	Follow-up duration	Survival/Outcome
1	Song et al. (2024)	Ruxolitinib	Responded	19.9 weeks	No	No	No	No	No	5.8 months	Alive
2	Wu et al. (2022)	Etoposide, DXM	Responded	NR	No	No	Yes	No	No	100 days	Alive
3	Guild et al. (2022)	Anakinra, corticosteroids	Responded	NR	No	NA	No	No	No	3 years	Alive
4	Schuelke et al. (2023)	NR	Responded	NR	No	NR	NR	No	No	6 months	Alive
5	Schuelke et al. (2023)	NR	No response	NR	NR	NR	NR	NR	NR	6 months	Dead

HLH, hemophagocytic lymphohistiocytosis; HSCT, hematopoietic stem cell transplantation; DXM, dexamethasone; CMV, cytomegalovirus; COVID-19, Coronavirus Disease 2019; mRNA, messenger ribonucleic acid; VZV, Varicella-Zoster Virus; NR, not reported; NA, not applicable; mg/kg, Milligrams per kilogram; yrs, years; wks, weeks.

Emapalumab was initiated as rescue therapy for refractory hyperinflammation unresponsive to standard treatments. Most patients had previously received high-dose corticosteroids and additional immunomodulators such as anakinra, cyclosporine, or intravenous immunoglobulin. Reported treatment regimens ranged from two to twenty doses (median seven), with treatment durations of one to 19 days (median twelve). None of the patients underwent hematopoietic stem-cell transplantation.

Clinical improvement was achieved in four of the five cases (80%), with resolution of fever, normalization of cytopenias, and biochemical recovery. One patient did not respond and died of progressive multiorgan failure. Surviving patients remained in remission (follow-up of three to 6 months). No adverse events or treatment-limiting toxicities were reported.

Overall, emapalumab was well tolerated and associated with favorable clinical outcomes in the majority of patients, supporting its therapeutic potential as salvage therapy for severe, treatment-refractory secondary HLH outside traditional etiologic categories.

## Discussion

This scoping review synthesizes all available real-world evidence on the use of emapalumab, a targeted interferon-γ–blocking antibody, across the full spectrum of hemophagocytic lymphohistiocytosis (HLH) and related hyperinflammatory syndromes. A total of 31 publications encompassing 86 unique patients were identified through comprehensive searches of PubMed, Scopus, and Web of Science. Reported cases spanned familial/genetic HLH, rheumatology-associated macrophage activation syndrome (MAS), malignancy-associated HLH, infection-associated HLH, CAR-T/IEC-HS–related HLH, and other secondary or idiopathic forms.

Through studies, emapalumab was most often employed as rescue therapy in patients with refractory or relapsing hyperinflammation unresponsive to conventional regimens, including corticosteroids, etoposide, and other cytokine-directed agents. Evidence consistently demonstrated rapid and sustained disease control, typically within one to 3 weeks of treatment initiation. In familial HLH, emapalumab effectively suppressed cytokine-driven inflammation and served as a bridge to hematopoietic stem-cell transplantation (HSCT) with excellent survival outcomes. In secondary forms—including rheumatologic, infectious, malignancy-related, and CAR-T–associated HLH—emapalumab similarly induced marked clinical and biochemical improvement in most cases, with treatment discontinuation rarely required. Importantly, treatment was well tolerated across all disease contexts. Reported adverse events were uncommon, generally mild, and seldom attributed to emapalumab. Mortality, when observed, was primarily related to underlying disease progression or multiorgan failure rather than drug toxicity.

In familial or genetically confirmed HLH, outcomes closely mirrored pivotal clinical trial data, demonstrating that interferon-γ blockade provides rapid cytokine suppression and disease stabilization prior to hematopoietic stem-cell transplantation (HSCT). High remission and survival rates reflected effective control of hyperinflammation, enabling timely HSCT and improved long-term survival. Interestingly, the incidence of severe infections was lower in real-world settings compared with clinical trials (Locatelli et al., 2020). Across pediatric series, adverse events were infrequent and generally mild, supporting the drug’s favorable safety profile in young children.

Prospective clinical trial in MAS associated with Still’s disease have demonstrated that emapalumab achieves durable disease control following glucocorticoid failure, with consistent improvements in fever, cytopenias, ferritin, and transaminase levels under a dosing regimen aligned with the HLH program (Benedetti et al., 2023). On 27 June 2025, the U.S. FDA the indication for emapalumab to include HLH/MAS in patients with known or suspected Still’s disease—encompassing both sJIA and AOSD—who exhibit inadequate response or intolerance to glucocorticoids, or recurrent MAS. This approval codified off-label practices already adopted in many centers and underscored the mechanistic rationale for interferon-γ blockade in MAS.

Importantly, clinical trial data (Benedetti et al., 2023) reported no opportunistic or atypical infections, despite suppression of interferon-γ activity. Herpes zoster infections, common in individuals with defective IFN-γ signaling, were not observed, likely due to per-protocol acyclovir prophylaxis. No infections caused by *Mycobacterium*, *Histoplasma capsulatum*, *Shigella*, *Salmonella*, *Campylobacter*, or *Leishmania* occurred, and bacterial or opportunistic infections were absent. Only mild viral infections or transient positive viral tests were reported, all resolving spontaneously or with standard treatment. These findings align with our real-world observations, where no opportunistic or severe infections were documented, even in heavily immunosuppressed patients receiving concomitant therapies. This concordance between trial and real-world data reinforces the selective immunomodulatory profile of interferon-γ blockade—attenuating hyperinflammation without compromising essential antimicrobial defense.

In malignancy-associated HLH, therapeutic benefit from emapalumab appeared most pronounced in patients with inflammation-predominant disease rather than those with extensive or rapidly progressive tumor burden (Johnson et al., 2024). The reported 24% response rate in malignancy-associated HLH warrants cautious interpretation. Outcomes within this subgroup were disproportionately influenced by the Johnson et al. cohort, in which 14 of 15 patients died, substantially weighting the overall estimate. Mortality across published cohorts was largely driven by malignant progression and multiorgan failure rather than clear evidence of drug-related toxicity. Notably, emapalumab was frequently administered in late-stage or salvage settings in patients with advanced, refractory malignancy and severe organ dysfunction, which likely confounds assessment of intrinsic therapeutic efficacy. While formal sensitivity analysis is beyond the scope of this scoping review, exclusion of the Johnson cohort would meaningfully alter the aggregate response rate, suggesting that the observed low response may reflect disease burden and timing of intervention rather than lack of biological activity. Collectively, these observations underscore the importance of early recognition and treatment during the hyperinflammatory phase—prior to irreversible organ injury or overwhelming neoplastic infiltration—to potentially optimize the therapeutic window for interferon-γ blockade in malignancy-associated HLH.

For infection-associated HLH, particularly EBV-driven disease, emapalumab produced favorable responses and often enabled transition to HSCT once inflammation was controlled. Reports consistently describe reassuring safety, with no evidence of impaired pathogen clearance or exacerbation of infection (Zhu et al., 2025). Mechanistically, blockade of interferon-γ likely mitigates the cytokine storm component of EBV-associated HLH without compromising host defense. Adverse events were infrequent and typically mild, such as transient mucosal bleeding or self-limited viral reactivation.

In the context of CAR-T–CRS and IEC-HS, clinical observations reinforce the mechanistic overlap between cytokine toxicity and HLH-like hyperinflammation. Rapid improvement after one or few doses of emapalumab has been reported in cases refractory to corticosteroids, tocilizumab, or anakinra (Schuelke et al., 2023). These data highlight the drug’s value as a rescue therapy in severe cytokine-driven complications of cellular immunotherapy. Safety data in this setting are likewise favorable, with reported complications attributed to the underlying inflammatory syndrome rather than drug toxicity. However, the current literature supporting emapalumab use remains limited—largely derived from a small pediatric pHLH cohort and scattered case reports, as summarized in our review results. In addition, the high cost and logistical challenges of procurement in urgent settings may restrict timely access. Evidence in adults, particularly those with IEC-HS, is extrapolated from pediatric and pre-clinical data, and its application continues to generate debate. Should elevations in interferon-γ be confirmed as a defining feature of IEC-HS, the targeted use of emapalumab could represent a rational therapeutic option ideally guided by patient-specific disease dynamics, biomarker profiles, and institutional experience.

In other secondary or idiopathic forms of HLH, early reports suggest that emapalumab can successfully control hyperinflammation in undefined or mixed-trigger cases, achieving remission with acceptable tolerability. Although data remain limited, these findings point to a potential broader therapeutic role for interferon-γ blockade in different HLH settings.

The conclusions of this review should be interpreted with caution given several inherent limitations in the available evidence. The literature on emapalumab in HLH is largely restricted to case reports and small case series with variable methodological rigor and inconsistent reporting quality. Adverse events were not systematically captured, and attribution is particularly challenging in critically ill patients receiving multiple concomitant immunosuppressive therapies. The absence of control groups limits causal inference regarding efficacy and precludes meaningful comparison with other cytokine-directed agents. Publication bias may further distort the evidence base, as unfavorable or complicated cases are less likely to be reported. The exclusion of non-English studies and conference abstracts introduces potential selection bias, particularly in a rare disease where geographically diverse case reports may meaningfully contribute to understanding. Substantial heterogeneity in dosing strategies, timing of initiation (including frontline versus salvage use), and concomitant therapies complicates cross-study comparisons and interpretation of response kinetics. Additionally, follow-up durations were often brief, limiting assessment of long-term survival, relapse risk, and delayed toxicities. In secondary HLH, the lack of standardized diagnostic frameworks—particularly in infection- and malignancy-associated subtypes—adds further variability that may influence outcome reporting. Collectively, these constraints indicate that real-world experience with emapalumab remains exploratory and underscore the need for prospective, systematically collected data to better define its role across HLH subtypes.

Despite these challenges, this review represents the first comprehensive synthesis of published real-world data on emapalumab across the full clinical spectrum of HLH. By aggregating evidence, it provides an inclusive and balanced overview of global clinical experience. Categorizing results by disease context—familial, rheumatologic, infectious, malignancy-associated, CAR-T/IEC-HS–related, and idiopathic HLH—enabled clearer recognition of consistent patterns in efficacy and safety across diverse etiologies. The inclusion of both pediatric and adult cases broadens the generalizability of findings and offers clinicians practical insights into real-world outcomes. Most notably, the reproducible rapid disease control and favorable tolerability observed across studies reinforce the translational consistency of interferon-γ blockade as a mechanistically targeted intervention in HLH.

Future research should prioritize the establishment of prospective, multicenter registries to capture standardized data on dosing, treatment sequencing, and long-term outcomes. Controlled comparative studies with other cytokine inhibitors—such as anakinra or ruxolitinib—are essential to define optimal therapeutic positioning and combination strategies. Incorporating biomarker-guided approaches, including interferon-γ gene signatures and CXCL9 kinetics, may further refine patient selection and real-time response assessment. Efforts should also focus on earlier initiation of emapalumab—before irreversible organ injury develops—particularly in infection- and malignancy-associated HLH. Longitudinal studies are warranted to characterize relapse dynamics, infection risk, and immune reconstitution following IFN-γ blockade. Finally, expanding investigation into adult and malignancy-driven HLH, currently underrepresented in clinical research, will be critical to optimizing the scope, timing, and integration of emapalumab within evolving HLH treatment algorithms.

## Conclusion

This scoping review integrates all available real-world evidence on emapalumab use in both familial and secondary HLH. Across diverse etiologies, interferon-γ blockade consistently achieved rapid and sustained control of hyperinflammation, enabling hematopoietic stem-cell transplantation in primary disease and remission in refractory secondary forms—including rheumatologic, infectious, malignancy-associated, and CAR-T/IEC-HS–related HLH. The favorable safety profile observed in both pediatric and adult populations supports the selectivity of IFN-γ inhibition in attenuating cytokine storm without compromising antimicrobial defense. Although current data are largely limited to small case series and reports, the accumulated experience positions emapalumab as a mechanistically targeted and well-tolerated option for severe, treatment-refractory HLH. Future prospective, biomarker-driven studies and standardized registries are needed to refine patient selection, optimize timing of initiation, and clarify its long-term role within evolving HLH management frameworks.

## Data Availability

The original contributions presented in the study are included in the article/supplementary material, further inquiries can be directed to the corresponding author.
